# PointAS: an attention based sampling neural network for visual perception

**DOI:** 10.3389/fncom.2024.1340019

**Published:** 2024-05-02

**Authors:** Bozhi Qiu, Sheng Li, Lei Wang

**Affiliations:** ^1^School of Electronic Information, Xijing University, Xi’an, China; ^2^Shaanxi Huanghe Group Co, Ltd., Xi’an, China

**Keywords:** PointAS, point cloud data, neural network, ata sampling, attention

## Abstract

Harnessing the remarkable ability of the human brain to recognize and process complex data is a significant challenge for researchers, particularly in the domain of point cloud classification—a technology that aims to replicate the neural structure of the brain for spatial recognition. The initial 3D point cloud data often suffers from noise, sparsity, and disorder, making accurate classification a formidable task, especially when extracting local information features. Therefore, in this study, we propose a novel attention-based end-to-end point cloud downsampling classification method, termed as PointAS, which is an experimental algorithm designed to be adaptable to various downstream tasks. PointAS consists of two primary modules: the adaptive sampling module and the attention module. Specifically, the attention module aggregates global features with the input point cloud data, while the adaptive module extracts local features. In the point cloud classification task, our method surpasses existing downsampling methods by a significant margin, allowing for more precise extraction of edge data points to capture overall contour features accurately. The classification accuracy of PointAS consistently exceeds 80% across various sampling ratios, with a remarkable accuracy of 75.37% even at ultra-high sampling ratios. Moreover, our method exhibits robustness in experiments, maintaining classification accuracies of 72.50% or higher under different noise disturbances. Both qualitative and quantitative experiments affirm the efficacy of our approach in the sampling classification task, providing researchers with a more accurate method to identify and classify neurons, synapses, and other structures, thereby promoting a deeper understanding of the nervous system.

## 1 Introduction

In recent years, the swift progress of 3D sensing technology has made acquiring and analyzing 3D data more accessible and garnered increasing attention. The point cloud, which serves as a foundational representation of 3D data, encompasses an un-structured collection of points delineating the geometry of an object. As the first-hand data captured by LiDAR or depth cameras, point clouds find extensive application in diverse fields including robotics, scene reconstruction, autonomous navigation in driving, virtual reality (VR) and human brain nervous system.

Nevertheless, a point cloud often comprises a substantial volume of data points, which can pose challenges for direct processing. The human brain’s unparalleled ability to navigate complex sensory data is a result of its intricate neural network, which efficiently encodes, processes, and decodes information. As Peter Dayan elucidates in “Theoretical Neuroscience” (Dayan and Abbott, 2005), neurons employ selective attention mechanisms to prioritize certain inputs over others, enhancing the processing of behaviorally relevant stimuli while filtering out noise or less critical information. Similarly, as outlined in “Vision” (Marr, 2010) by Marr, David, our visual system uses sophisticated strategies to segment and interpret the visual field, focusing on key features that aid in object recognition and scene understanding. Hence, downsampling operations are typically used to decrease the point cloud’s size and enhance computational efficiency. Point cloud downsampling is a technique employed in computer vision and robotics to reduce the size of a point cloud dataset while retaining its crucial features. This is important because large point cloud datasets can be computationally expensive to process, making downsampling a crucial step in many applications. The objective of downsampling is to eliminate redundant points from the dataset while preserving the overall shape and structure of the represented object or scene.

Classical sampling methods like random sampling (RS) (Eldar et al., 1997) and farthest point sampling (FPS) (Moenning and Dodgson, 2003). The FPS algorithm is recognized as an efficient method for point cloud sampling, employing an iterative approach to select points furthest from the current selection. This strategy enables comprehensive sampling within a shorter timeframe, making FPS particularly suitable for large-scale point cloud data. Its efficiency allows it to generate relatively uniform sampling outcomes more quickly compared to alternative methods. However, while FPS ensures spatial distribution and representativeness by selecting the farthest point from the existing set, it lacks consideration for the specific downstream task. Consequently, it may select non-informative points relevant to the task. For instance, while Random Sampling (RS) is effective, it may overlook sparse regions due to its random selection approach. On the other hand, while FPS provides greater coverage of the entire point dataset, it faces a latency bottleneck in parallel computation. Thus, these conventional methods fail to consider the subsequent processing of the sampled points, potentially resulting in the selection of irrelevant data for the down-stream task, thereby leading to subpar performance. In contrast to non-learned methods like FPS, deep learning has found extensive application in processing intricate point cloud data. Consequently, in recent years, researchers have introduced various differentiable downsampling techniques within deep learning frameworks (Shi et al., 2015; Liu and Kang, 2017; Shao et al., 2019; Yang et al., 2019; Lang et al., 2020; Nezhadarya et al., 2020; Yan et al., 2020; Zong et al., 2021). Within this body of work, one category of methods focuses on optimizing sampling outcomes for downstream tasks, exemplified by SampleNet (Lang et al., 2020) and S-NET (Shao et al., 2019). Another category aims to enhance the sampling module in existing methods to mitigate challenges like noise and outliers in point cloud applications, as demonstrated by PointASNL (Yan et al., 2020) and ASHF-Net.

S-NET and SampleNet highlight the significant advancements attainable through task-specific sampling techniques, demonstrating how these networks can improve sampling efficiency. These approaches leverage limited sampling data to optimize downstream task performance, consistently outperforming traditional task-independent methods across various applications. However, S-NET, while excelling in classification and geometric reconstruction, relies on sampling algorithms like FPS that do not consider the subsequent task, limiting their adaptability during network training. Conversely, SampleNet implements a differentiable point cloud sampling method. But it may struggle with incorporating meaningful points for severely under-sampled structures, failing to account for global geometric properties.

To address the need for considering both global and local point cloud properties while ensuring a feasible sampling process, we introduce PointAS, an end-to-end downsampling neural network. Drawing inspiration from the foundational principles of neural science and human vision, PointAS capitalizes on the mechanisms of selective attention and feature prioritization to effectively tackle the challenges posed by point cloud data. The human brain employs neurons that enhance certain inputs while filtering out noise or less critical information, optimizing the processing of behaviorally relevant stimuli. Similarly, our adaptive sampling module reweights the surrounding neighbors of initial sampling points obtained through farthest point sampling (FPS), allowing for adaptive migration in the sampling results, thereby focusing on the most informative features and disregarding redundant data.

Moreover, the human visual system provides another layer of sophistication to our method. It integrates global context with local details, using foveal vision for high-resolution and peripheral vision for wide-field recognition. Analogously, the attention module in PointAS aggregates global features with the input point cloud data while enabling the extraction of local features. This dual focus on global and local properties enhances the classification performance, much like how the human eye can accurately identify objects within a scene by combining central detail with a broader perspective.

PointAS overcomes these limitations by considering both global and local properties of the point cloud during the sampling process. It achieves sequential sampling similar to FPS but maintains significant task-oriented results, even with noisy and density-variable inputs. The effectiveness of PointAS hinges on its adaptive sampling module and attentional sampling module, which work in tandem to select the most informative points while discarding redundant data. To summary, our key contributions are as follows:

1. We present PointAS, an end-to-end point cloud downsampling network based on an attention mechanism, drawing from principles in neural science and human vision to find key points in large datasets, enhancing downstream classification tasks.
2. PointAS can be jointly trained with multiple sample sizes to produce a single compact model that can generate samples of arbitrary length and is robust to noisy inputs for outstanding performance.
3. Good qualitative and quantitative results are obtained on common point cloud benchmarks, demonstrating the effectiveness of the proposed sampling method.

## 2 Related work

### 2.1 Deep learning on point clouds

With the notable success of Convolutional Neural Networks (CNNs) and trans-formers in the domains of computer vision and natural language processing, there has been a growing interest among researchers in extending these methodologies to process 3D point cloud data. However, it is important to note that some of the techniques commonly employed in image processing may not be readily applicable to point cloud data due to its irregular and sparse nature. Consequently, specialized deep learning methods tailored for 3D point clouds are imperative. Deep neural networks have found widespread application in the analysis of point cloud data, encompassing tasks such as point cloud classification, generation, alignment, segmentation, geotagging, and automatic coding. In the early stages of this research, the focus was on conventional data representation in the form of 3D voxels. These approaches involve decomposing the point cloud into uniform voxels in three-dimensional space, utilizing a predetermined resolution, and subsequently applying volume convolution techniques.

In recent times, considerable research efforts have been directed toward the development of innovative local aggregation operators tailored for point clouds. The primary objective is to minimize the loss of intricate details while processing point sets efficiently. Notably, PointNet (Qi et al., 2017a) incorporates a multilayer perceptron (MLP) that maps each point’s data from its coordinate space to a high-dimensional feature space. This information is then amalgamated into a representative feature vector through global pooling, ultimately determining the class of the input point cloud via a fully connected layer (FC) mapping. PointNet stands as a pioneering approach in directly processing raw point clouds. Building on this foundation, PointNet++ (Qi et al., 2017b) extends the framework with a layered neural network architecture capable of extracting both global and local features. It excels particularly in handling point clouds with varying densities. PointCNN (Li et al., 2018) introduces a deep learning framework specialized for point cloud classification and segmentation, achieving commendable results in these tasks. Its key strength lies in the utilization of a novel convolutional algorithm adept at efficiently performing operations on point clouds. Similarly, DGCNN (Phan et al., 2018) provides a tailored deep learning framework for point cloud classification and segmentation. It efficiently extracts both global and local features from point cloud data through the use of a dynamic graph construction method based on adjacency graphs. Finally, Point Transformer (Zhao et al., 2021) is designed for point cloud classification and segmentation, incorporating an attention mechanism in the Transformer model to efficiently capture point cloud features. Despite the rapid advancements in point cloud technology across various fields, the application of downsampling techniques remains relatively limited.

### 2.2 Point cloud sampling

Given the inherent challenge of processing high-resolution dense point clouds, it becomes imperative to engage in point cloud sampling or simplification. Consequently, a range of methodologies has been explored to streamline dense point cloud data.

Random Sampling (RS) stands as a widely employed method for unsupervised sampling. In RS, the fundamental assumption is that each point within the original point cloud data carries an equal probability of selection. This method randomly selects the desired number of points from the initial point cloud data. RS operates with re-markable speed, as it does not necessitate point-to-point distance computations or any supplementary calculations during the sampling process. However, a drawback of RS lies in its indiscriminate selection, where all points in the point cloud are chosen with uniform probability. This leads to a dearth of object shape information and renders it sensitive to the presence of noisy points. It stands out for its minimal computational overhead, but it can be susceptible to issues of density imbalance.

Farthest Point Sampling (FPS) represents the most prevalent downsampling approach in the realm of point cloud analysis. FPS achieves broad coverage of the input point cloud by iteratively selecting points that are farthest from each other. However, it is essential to note that FPS operates solely within the Euclidean space and does not factor in any subsequent processing of the sampled points. This renders it susceptible to noise and agnostic to specific tasks, potentially leading to suboptimal performance.

To mitigate the limitations of Farthest Point Sampling (FPS), several deep learning-based methodologies have been put forth. Notably, a recent study by Critical Point Net (CP-Net) (Xu et al., 2024) endeavors to achieve deterministic sampling outcomes. This approach assesses the significance of points based on their contribution to the global maximum pool of features. Key points are extracted and preserved in subsequent layers, while less influential points are discarded. However, it is worth noting that the sampled points constitute only a subset of the original input data and may be subject to distortion in the presence of noise and outliers.

Much like CP-Net, S-NET considers the contribution of sampled points to a specific task by initially selecting data points for subsequent tasks. To maintain similarity with the original input, each selected point is paired with its nearest neighbor. Nevertheless, due to the non-trivial nature of this matching step, the accuracy of the sampled points is inevitably compromised. To address this challenge, SampleNet introduces a soft projection mechanism based on S-NET. Through optimization of the weights in this projection, the matching of sample points can be approximated as a nearest-neighbor se-lection, thereby rendering the matching step exceedingly precise.

Recent studies conducted by S-NET and SampleNet have illustrated that generating a set of simplified points optimized for a downstream task leads to more effective sampling outcomes. Furthermore, these simplified points correspond to a subset of the initial data, thereby reducing training losses. Both methodologies approach the sampling process as a generative task, generating all points in a single iteration, yet this approach may not adequately address sample dependency, potentially leading to suboptimal outcomes. In this study, we amalgamate and implement the FPS algorithm within the adaptive downsampling module, while incorporating the proximity trans-former module to assimilate local data information. Within the Attention module, we leverage PointNet as our feature extraction network for capturing global information. Finally, an attention-based time series model is integrated to derive the ultimate downsampled data.

## 3 Methodology

**Figure 1:**
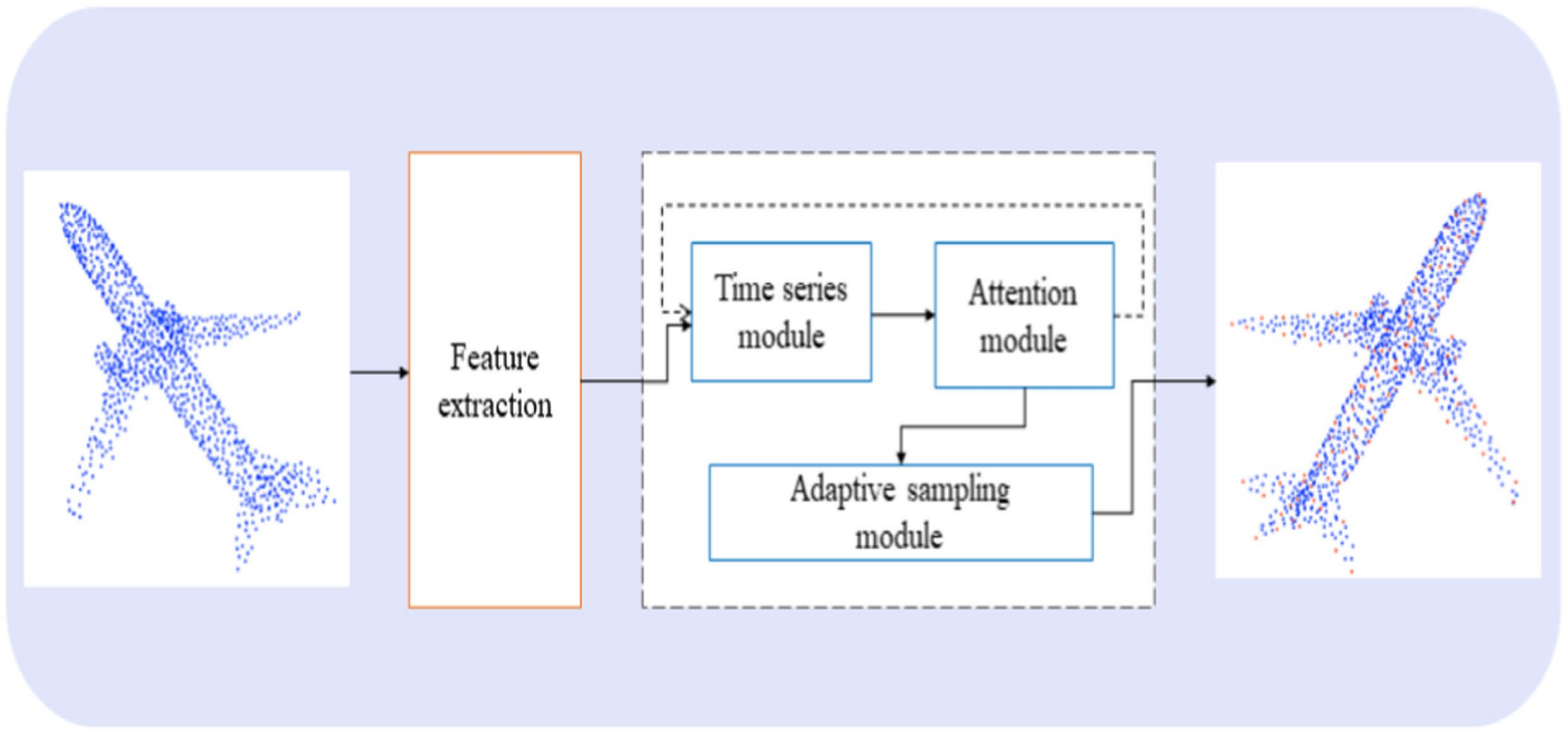
An overview of PointAS.

Figure 1 provides an overview of our sampling method, PointAS, comprising two primary components: (a) The Adaptive Sampling Module for dynamic position adjustment, and (b) The Attention Sampling Module for sequential point selection.

Initially, the input point cloud data undergoes processing by the input time series model, followed by an iterative step within the attention module to compute the downsampled point cloud. Through the attention mechanism, this step effectively integrates global feature information for subsequent modules, surpassing the capabilities of traditional algorithms. Subsequently, the sampling points based on the global feature information are inputted into the adaptive sampling module to obtain adaptive downsampling points. These points are then subjected to feature extraction through the feature extraction network to extract local features. The final output sampling points are a fusion of both global and local feature information. Unlike traditional algorithms that typically focus solely on high-frequency edge information or low-frequency planar information, the method proposed in this paper excels in extracting and combining multi-level high-dimensional features of the point cloud. In this section, we provide a detailed explanation of how these components operate and their implications for downstream tasks.

Let us assume the input to the PointAS is an unordered point cloud $P={\left\{p_{i}\in R^{3}\right\}}_{i=1}^{n}$ with *n* points, with $p_{i}=\left(x_{i},y_{i},z_{i}\right)$ representing Specific values in 3D axes. $Q={\left\{q_{i}\in R^{3}\right\}}_{j=1}^{m}$ and $R={\left\{r_{i}\in R^{3}\right\}}_{k=1}^{f}$ respectively denote the result of the adaptive sampling module and the final downsampling points. *m* is the number of points obtained by adaptive sampling and f is the number of final results with n>m>f . In order to improve the resulting efficiency of downsampling, R denotes a generated point cloud of f points that may not be a subset of P . Moreover, let $F_{\theta }\left(\cdot \right)$ denote PointAS with the parameters θ , which describe the process of turning P to Q . PointAS is trained with two loss terms:


(1) $$L^{ds}=L_{\mathrm{task}}\left(R\right)+\alpha \times L_{sim}\left(R,P\right)$$

(2) $$R=F_{\theta }\left(P\right)$$

(3) $$L_{\mathrm{task}}\left(R\right)=-\frac{1}{f}\overset{f}{\underset{j}{\sum }}\overset{c}{\underset{i=1}{\sum }}z_{ij}\times \log\left(v_{ij}\right)$$

Equation 1 represents the total loss function, which consists of two terms: the task-oriented loss $L_{\mathrm{task}}\left(R\right)$ and the similarity loss $L_{sim}\left(R,P\right)$ . Here, as described in Eq. 2, *R* represents the output of the downsampling network $F_{\theta }\left(P\right)$ , where *P* is the input point cloud. The coefficient α serves as a moderating term to balance the importance of the two optimization objectives and typically takes the value of 1.

The task-oriented loss $L_{\mathrm{task}}\left(R\right)$ aims to optimize the sampled set *R* to perform well on the downstream task, typically classification. It is computed using the cross-entropy function, as described in Eq. 3. In this equation, *c* represents the total number of classification categories, $z_{ij}$ denotes the ground truth label value of class *i* for point $R_{j}$ , and $v_{i}$ denotes the predicted probability of belonging to class *i* for point $R_{j}$ . The summation is performed over all points j in the sampled set *R* and all classes *i*.

The second term in Eq. 1, $L_{sim}\left(R,P\right)$ , is designed to encourage the simplified point cloud *R* to be closer to the input point cloud *P*. This term provides a form of regularization to control the range of the generated point cloud. The specific formula for $L_{sim}\left(R,P\right)$ is given in Eq. 9, which will be elaborated further below. The coefficient α serves as a moderating term to balance the importance of the two optimization objectives and typically takes the value of 1.

Overall, Eq. 1 combines the task-oriented loss and the similarity loss to guide the downsampling network to produce compact and task-relevant point clouds while ensuring their fidelity to the original input. This formulation helps in preserving task performance with the sampled point clouds while also maintaining their structural integrity.

### 3.1 Adaptive sampling module

**Figure 2:**
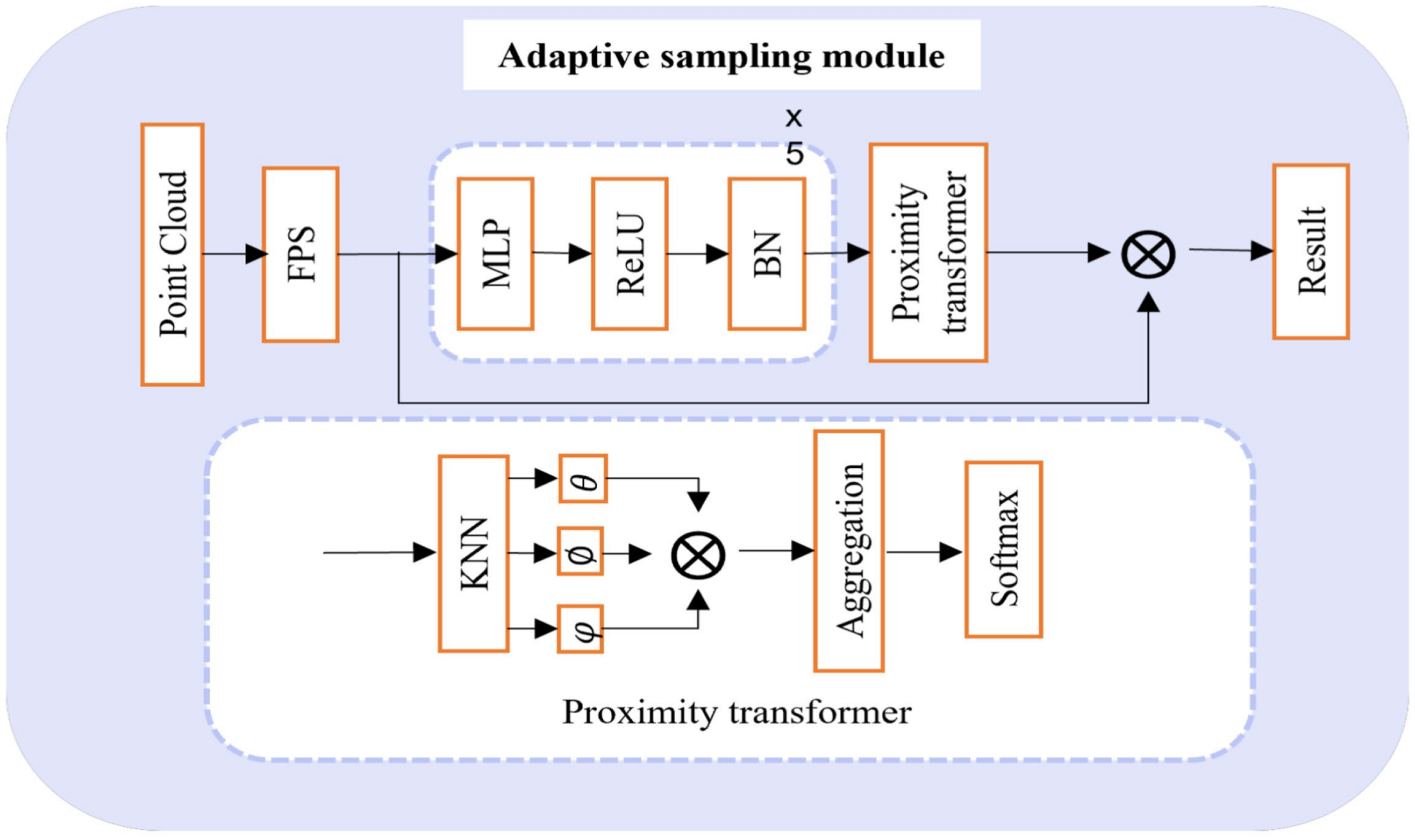
Adaptive sampling module.

The concrete implementation process of this module is illustrated in Figure 2. Here, the initial step involves the utilization of the Farthest Point Sampling (FPS) algorithm. This algorithm is chosen for its capacity to generate relatively uniform sampled points. Through FPS, the input point cloud data is preliminarily downsampled to yield key point data. However, it is important to acknowledge two primary challenges associated with FPS: (1) It exhibits high sensitivity to outlier points, rendering it notably unstable when confronted with real-world point cloud data. (2) The sampled points derived from FPS must necessarily form a subset of the original point clouds, which can pose difficulties in reconstructing the original geometric information in instances of occlusion and missing data errors during acquisition.

Following this, a K-Nearest Neighbors (KNN) operation (Guo et al., 2003) is performed on each keypoint data to acquire the keypoint neighborhood of the input data. Since the attention mechanism of the neighborhood, based solely on location information, exclusively accounts for low-level distance information, it falls short of capturing the high-level feature relationships between points in the neighborhood. To address this limitation, we extract high-level feature information from the neighborhood by employing a straightforward mapping operation that takes the location information of the point cloud as input. Leveraging these high-level details in conjunction with the proximity transformer operation, we ultimately obtain subsampled data points with adaptive migration.

For this module, $P={\left\{p_{i}\in R^{3}\right\}}_{i=1}^{n}$ is the input of the module. After the FPS module, we use five simple MRB modules for its high-level feature extraction, $F_{p}\in R^{n\times d}$ is its high-level feature representation and d denotes its dimension. In proximity transformer operation, We start by finding k neighbors of each feature in the high-level feature space which is denoted as $\mathrm{Neighbor}\left(F_{i}^{p}\right)={\left\{F_{i1},\cdots ,F_{ik}\right\}}_{j=1}^{k}$ , then feature updating of group member $F_{i}^{p}$ can be written in Eq. 4


(4) $$F_{i}^{p}=\mathrm{Aggregation}\left(\begin{array}{l} R\left(F_{i}^{p},F_{ij}^{p}\right)*\theta \left(F_{i}^{p}\right),\\ \forall F_{ij}^{p}\in \mathrm{Neighbor}\left(F_{i}^{p}\right) \end{array}\right)$$

where a pairwise function $R\left(\cdot \right)$ computes a high-level relationship between group members, its specific expression can be described in Eq. 5


(5) $$R\left(F_{i}^{p},F_{ij}^{p}\right)=\mathrm{softmax}\left(\frac{\varphi \left(F_{ij}^{p}\right)*\emptyset \left(F_{ij}^{p}\right)}{\sqrt{d}}\right)$$

In the above expression, $\varphi \left(\cdot \right)$ and $\emptyset \left(\cdot \right)$ are independent two linear transformations. Once we have the updated features based on the neighborhood, using Softmax on them gives us the attention weight for each neighbor point calculated from the high-level features, described in Eq. 6. Therefore, the result of adaptive downsampling for each neighborhood is obtained, which can be expressed by Eq. 7:


(6) $$W_{i}^{p}=\mathrm{softmax}\left(F_{i}^{p}\right)$$

(7) $$Q_{i}={\left(W_{i}^{p}\right)}^{T}*\mathrm{Neighbor}\left(P_{i}\right)$$

### 3.2 Attention sampling module

**Figure 3:**
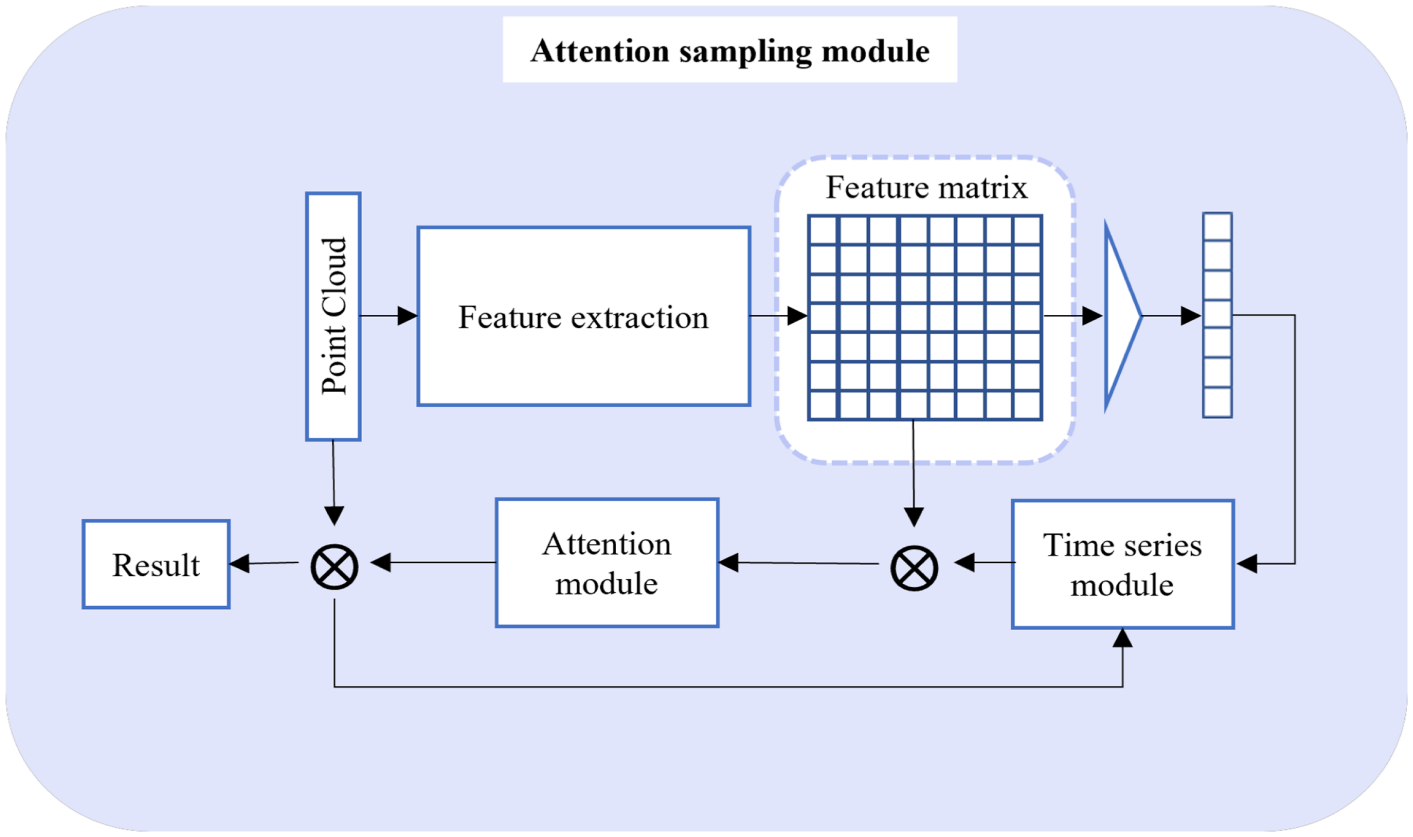
Attention sampling module.

The comprehensive architecture of the Attention Sampling Module is outlined in Figure 3. The input data for this module is the output produced by the Adaptive Sampling Module. In this context, to effectively leverage the global point cloud information, we treat the point cloud data as a distinctive sequence, implementing point cloud downsampling through a global attention operation. However, due to the substantial volume of point cloud data, while advanced sequence models may yield superior results, they come at the cost of a significantly expanded model parameter count. This inevitably hinders the computational efficiency of the overall point cloud downsampling process. Hence, in our pursuit of efficiency, we initiate the process by utilizing the feature extraction network to acquire high-level feature information from the input data. Following this, we obtain the weight matrix through the integration of the classical LSTM model with an attention operation. Ultimately, this leads to the derivation of the downsampled data, leveraging the comprehensive data insights.

The input of this module is $Q={\left\{q_{i}\in R^{3}\right\}}_{j=1}^{m}$ , which is the output of the adaptive sampling module. We use PointNet to extract global features $F_{q}\in R^{m\times d}$ , and then obtain the global feature vector $G^{q}\in R^{d}$ by the Max pooling operation. As mentioned above, we use a simple two-layer LSTM model for the timing calculation. By inputting the global feature vector into the time series model to obtain the dimension weight, and multiplying it with the feature matrix, the global-based downsampling point can be calculated in Eqs. 8 and 9.


(8) $$W^{q}=\mathrm{softmax}\left(\mathrm{LSTM}\left(G^{q},r_{i-1}\right)*F^{q}\right)$$

(9) $$r_{i}={\left(W\right)}^{T}*Q$$

The LSTM model has two inputs, one is the global feature vector, and the other is the downsampling point at the last time (initially set to 0). Then the downsampling point at the last moment was used as one of the inputs of the time series model to iterate and obtain the final downsampling point cloud result $R={\left\{r_{i}\in R^{3}\right\}}_{k=1}^{f}$ .

### 3.3 Loss function

Given a point cloud of $P={\left\{p_{i}\in R^{3}\right\}}_{i=1}^{n}$ , the goal is to generate a subset of $R={\left\{r_{i}\in R^{3}\right\}}_{k=1}^{f}$ which is optimized for the task T. Suppose that the objective function for this task is expressed as $\mu \left(\cdot \right)$ , our final ideal downsampling data can be given by Eq. 10:


(10) $$R^{*}=\underset{R}{\mathrm{argmin}}\mu \left(F_{\theta }\left(P\right)\right)$$

In order to encourage the second property in the loss function, a simplification loss is utilized in Eq. 11. Denoting average nearest neighbor loss (ANNL) and maximal nearest neighbor loss (MNNL) as:


(11) $$\begin{array}{l} L_{sim}\left(F_{\theta }\left(P\right),P\right)=\\ \frac{1}{\left|F_{\theta }\left(P\right)\right|}\underset{x\in F_{\theta }\left(P\right)}{\sum }\min_{y\in P}\|x-y\|{\;}^{2}+\beta *\max_{x\in F_{\theta }\left(P\right)}\min_{y\in P}\|x-y\|{\;}_{2}^{2} \end{array}$$

Where β acts as a moderating term coefficient usually taken as one, which aims to train toward the MNNL constraints while sampling the ANNL that is satisfied during training.

## 4 Experiment

This section conducts an assessment of the performance and resource utilization of PointAS in the context of task-specific point cloud downsampling. PointAS is designed to serve as a versatile module that can seamlessly integrate with any point cloud processing framework necessitating downsampling. The primary focus of this study is to assess the model’s performance in the realm of point cloud classification.

In this experiments, we compare the proposed PointAS against a range of downsampling methodologies mentioned in the introduction, encompassing: (1) conventional and widely used approaches such as Farthest Point Sampling (FPS), random sampling, and voxel-based methods; and (2) task-centric techniques including APSNet (Liu et al., 2022) and S-NET. Additionally, we incorporate downsampling approaches utilizing differentiable relaxation matching processes, such as SampleNet. All experiments are executed on an RTX A5000 (24GB), and during the training of PointAS, we employ the Adam optimizer with an initial learning rate of 0.7. It is worth noting that the parameters of the downstream task network (PointNet) are held constant during the entire training process.

### 4.1 Dataset

The classification tasks are tested on the ModelNet40 (Van den Herrewegen and Tourwé, 2023) dataset. Which offers extensive category coverage with 40 different object categories such as airplanes, cars, chairs, etc. This dataset provides a diverse range of objects, making it suitable for various 3D visualization studies. Additionally, it comprises a large number of instances, including approximately 12,311 clean point cloud models, with 9,843 utilized for training and the remaining 2,468 for testing.

In our approach, we employ the coordinates of the point cloud as input without incorporating any additional attributes. For the classification task, we utilize PointNet as the designated network, trained on point clouds consisting of 1,024 points. Adopting a fixed sampling rate aligns more closely with practical requirements, and adjusting the sampling rates for different volumes of data can facilitate the development of diverse tasks. In this study, we employ a fixed sampling rate to examine the model’s performance under various rates. The sampling rate is defined as $\log_{2}\left(1,024/m\right)$ , where m represents the number of sampled points.

### 4.2 Model evaluation metrics

In deep neural network model training, the evaluation metrics commonly used include accuracy (ACC), and ROC, among others. These metrics are explained in detail below.

Table 1 illustrates the confusion matrix for a binary classification task. In this context, TP stands for true positive (meaning the instance is positive and predicted as positive), FP represents false positive (indicating the instance is negative but predicted as positive), FN denotes false negative (meaning the instance is positive but predicted as negative), and TN signifies true negative (indicating the instance is negative and predicted as negative). In binary classification, both TP and TN represent accurate predictions, allowing ACC to be calculated in Eq. 12:


(12) $$ACC=\frac{TP+TN}{TP+FP+FN+TN}\text{.}$$

**Table 1:** Confusion matrix for binary classification.

	Positive	Negative
True	True Positive (TP)	False Positive (FP)
False	False Negative (FN)	False Negative (TN)

The ROC curve is a graphical tool used to represent the performance of a classification model. It depicts the performance of the classifier under different thresholds by taking the True Positive Rate (TPR) and False Positive Rate (FPR) as horizontal and vertical coordinates, and the formula for calculating the two is shown in Eq. 13:


(13) $$TPR=\frac{TP}{TP+FN}\;FPR=\frac{FP}{FP+TN}$$

AUC (Area Under the ROC Curve) is the area under the ROC curve, which is used to measure the performance of the classifier. The closer to 1 the AUC value is, the better the performance of the classifier; conversely, the closer to 0 the AUC value is, the worse the performance of the classifier is. The closer the value is to 1, the better the performance of the classifier is; conversely, the closer the AUC value is to 0, the worse the performance of the classifier is. In practice, we often calculate the AUC value to evaluate the performance of the classifier.

## 5 Results

### 5.1 Model evaluation

**Figure 4:**
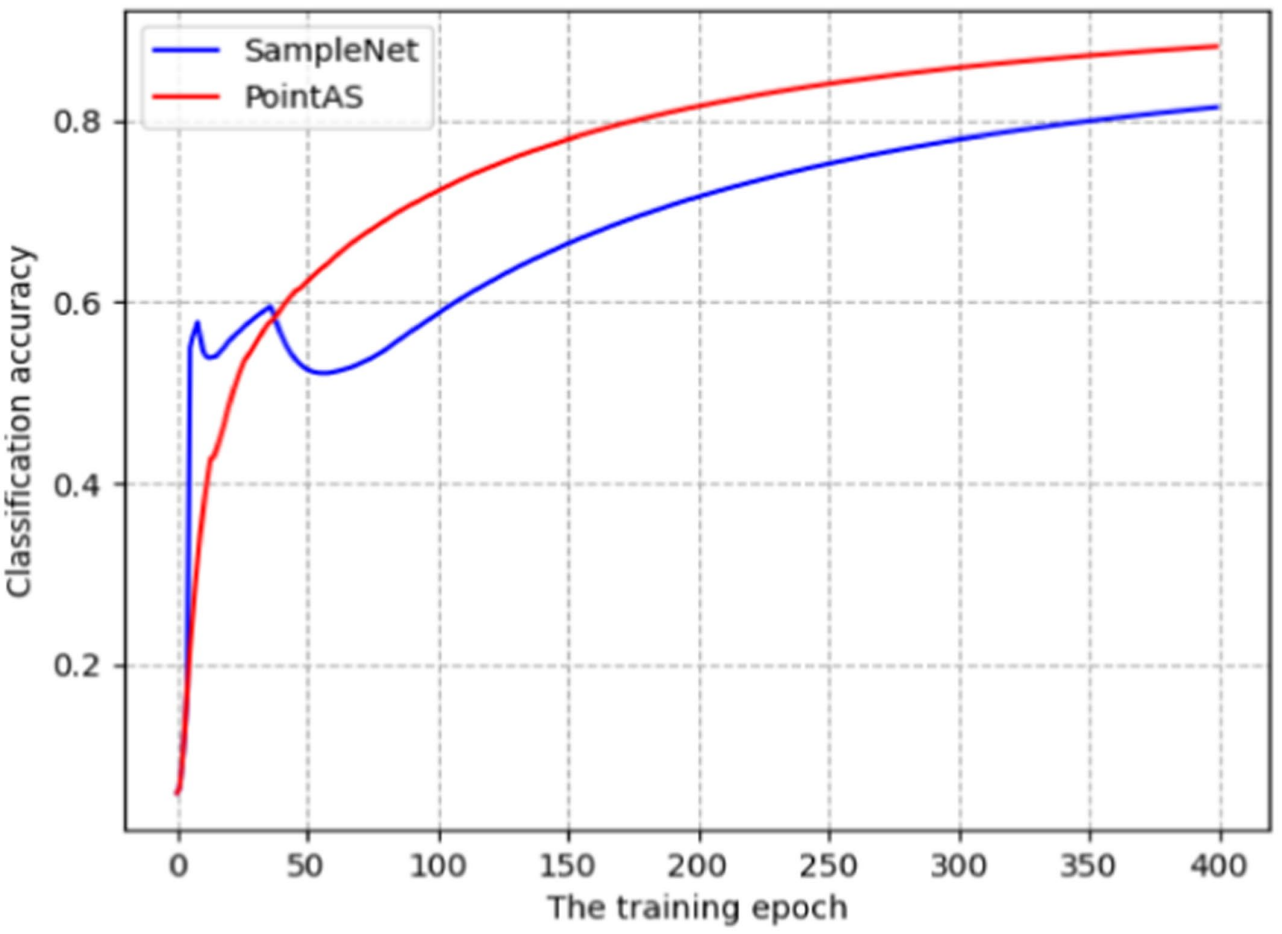
Accuracy of PointAS and SampleNet on ModelNet40 datasets.

The PointAS network undergoes training and evaluation using the ModelNet40 dataset. To provide a clearer perspective, we compare the training process with SampleNet, as depicted in Figure 4. The figure illustrates the classification accuracy over 400 epochs of iterations with a sampling ratio set at 5 (*m* = 128). The final results reveal that PointAS achieves an impressive accuracy of 87.02% for the classification task, surpassing SampleNet’s 82.75%. Notably, SampleNet demonstrates a faster growth in classification accuracy than PointAS in the initial 40 epochs of training. However, it experiences a decline in accuracy after the 8th and 38th epochs, possibly attributable to the optimization search of the SampleNet algorithm getting stuck in local optima. Post the 55th epoch, the accuracy of the SampleNet model gradually improves, but it consistently lags behind PointAS. The training curve of PointAS exhibits a smoother trajectory, with accuracy steadily increasing over the course of training. This underscores the superior robustness of the PointAS network. However, considering the resource utilization, the time required for PointAS to iteratively train the target dataset once is 132 s, which is slightly higher than SampleNet’s 109 s vs. APSNet’s 121.

### 5.2 Model comparisons

Table 2 provides a comprehensive quantitative comparison of various downsampling methods, including our proposed approach and the series of methods mentioned earlier. Initially, with a modest downsampling rate, conventional techniques like Random Sampling (RS) and Farthest Point Sampling (FPS) exhibit commendable performance, maintaining high accuracy even without extensive training data. Notably, FPS stands out, achieving an impressive accuracy of 88.34% at a downsampling rate of 1 (*m* = 512), surpassing both S-Net and SampleNet. The efficiency of the FPS algorithm stems from its iterative selection of points furthest from the currently chosen point, facilitating comprehensive sampling within a shorter timeframe. This attribute renders FPS particularly effective for sampling large-scale point cloud data, enabling it to generate relatively uniform sampling outcomes more rapidly than other sampling methods. However, as the downsampling rate increases, the limitations of traditional methods become evident. These methods can only downsample based on the original point cloud data and lack adaptability to meet the requirements of downstream tasks. This is particularly noticeable when the downsampling rate reaches 5 (*m* = 32) or higher, where the classification accuracy of FPS and RS drops to less than 30%.

**Table 2:** Classification accuracy with different downsampling methods.

*m*	RS	FPS	S-Net	APSNet	SampleNet	PointAS
512	87.52	88.34	87.80	88.78	88.16	89.26
256	77.09	83.64	82.38	88.46	84.27	89.10
128	56.44	70.34	77.53	84.04	82.75	87.02
64	31.69	46.42	70.45	82.11	80.86	82.02
32	16.35	26.58	60.70	81.56	80.31	81.94
16	7.15	13.29	36.16	80.26	79.09	80.07
8	3.27	3.47	20.81	78.37	70.94	75.37

Downsampling algorithms tailored to specific downstream tasks demonstrate notable classification accuracy even in the face of high downsampling rates, better aligning with practical needs. Table 2 highlights that both SampleNet and APSNet consistently achieve classification accuracies above 70% across various sampling scenarios. At a downsampling rate of 1 (*m* = 512), the accuracy soars to 88%. Remarkably, even at a sampling rate of 4 (*m* = 64) or higher, accuracy remains around 80%, signifying a significant enhancement in sampling performance compared to traditional task-independent methods (RS, FPS).

In comparison to other downsampling approaches, the PointAS method proposed in this paper demonstrates notable accuracy improvements at specific downsampling rates. At a downsampling rate of 1 (*m* = 512), it achieves an accuracy of 89.26%, and this accuracy remains above 80% as the downsampling rate increases to 4 (*m* = 64). This is attributed to the proposed algorithm’s initial utilization of global information to generate downsampling points, followed by a local adaptive adjustment process to derive the final downsampling points. This approach excels particularly when the downsampling rate does not exceed 5 (*m* = 32). However, when the downsampling rate is exceedingly high, and the overall point cloud data becomes limited, the local integration of information and the local adaptive adjustment module face challenges, resulting in a downstream task performance that falls short of APSNet.

**Figure 5:**
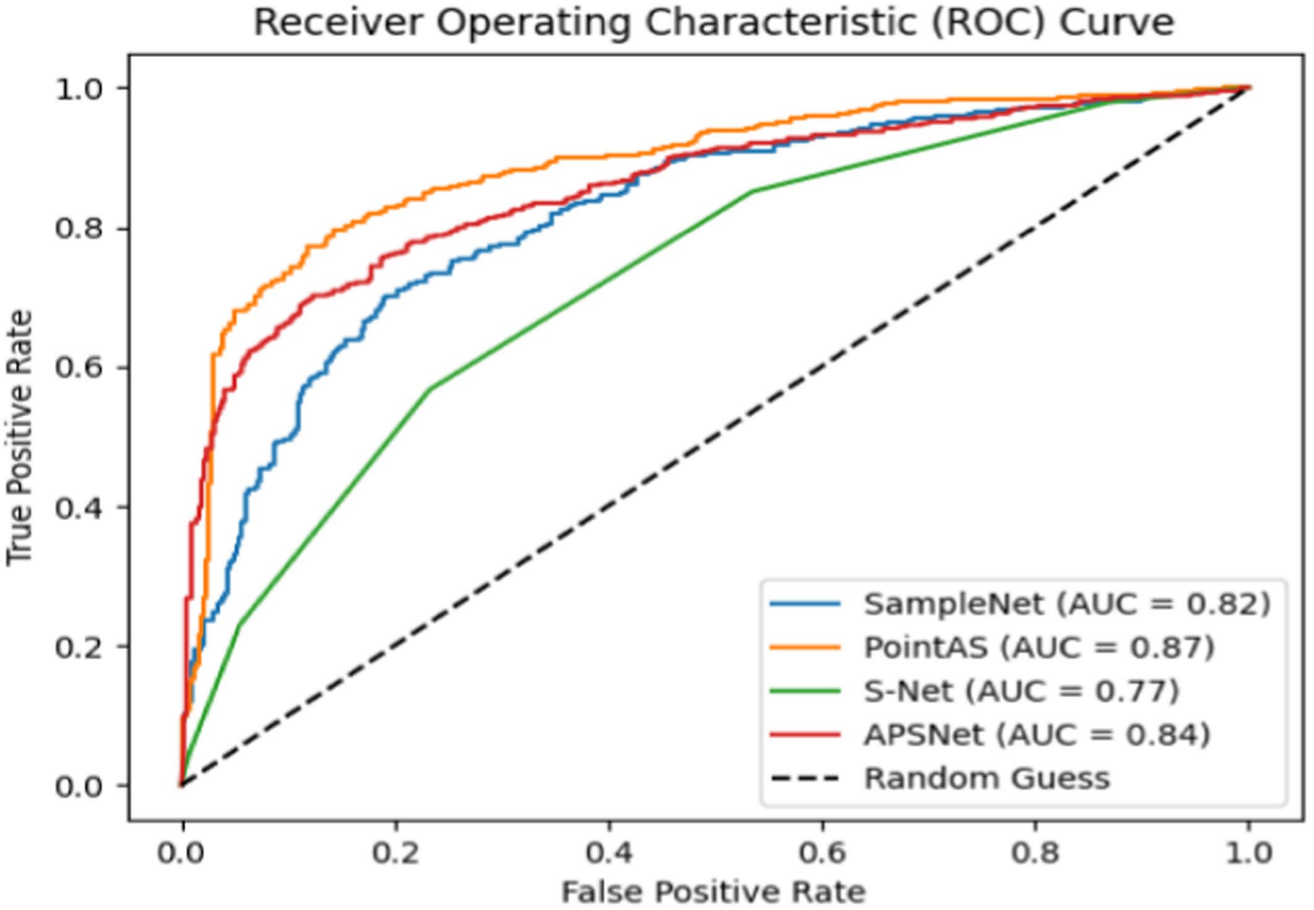
ROC of different methods on ModelNet40 datasets.

**Figure 6:**
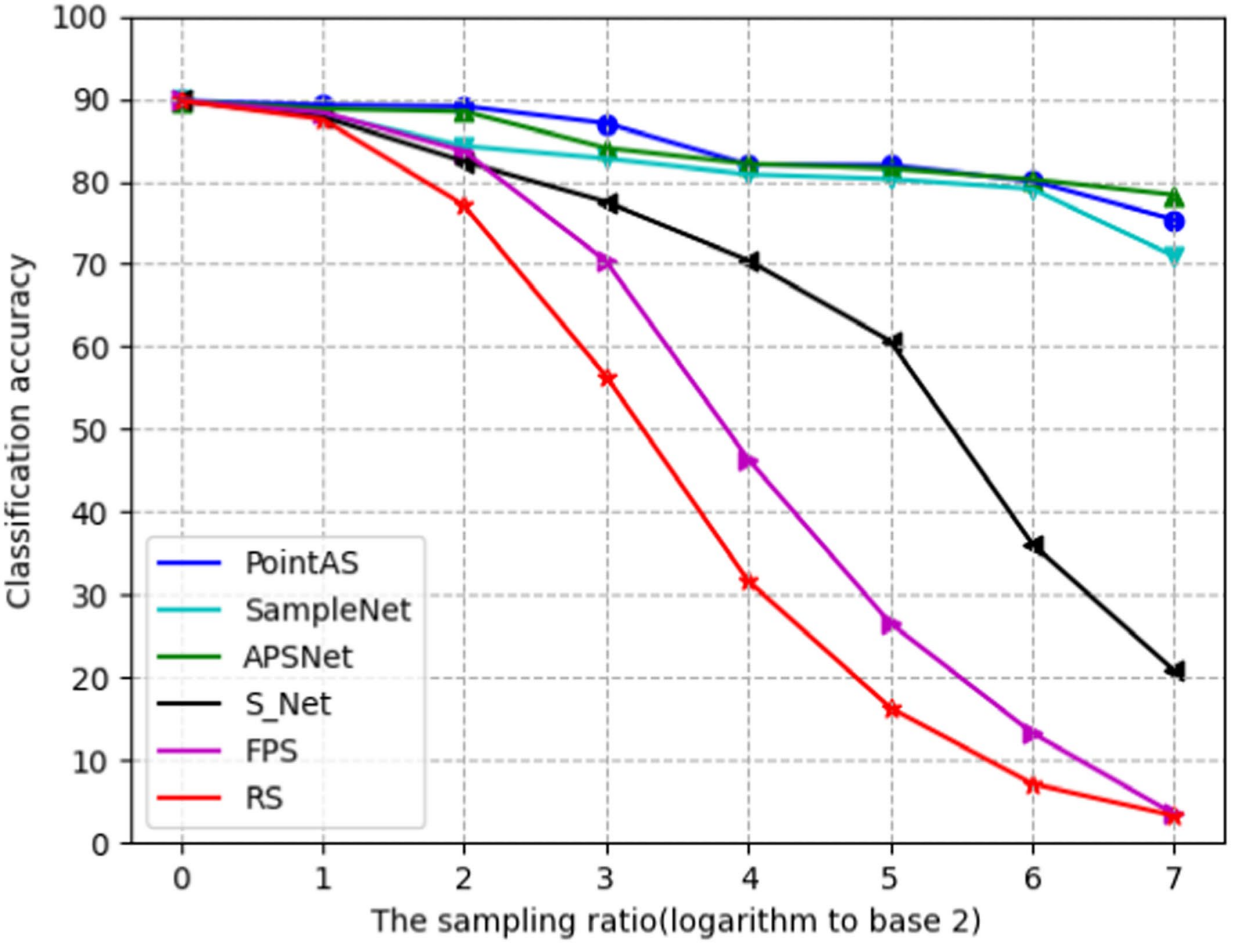
Evolution of classification accuracy as a function of sample ratio for six sampling methods.

**Figure 7:**
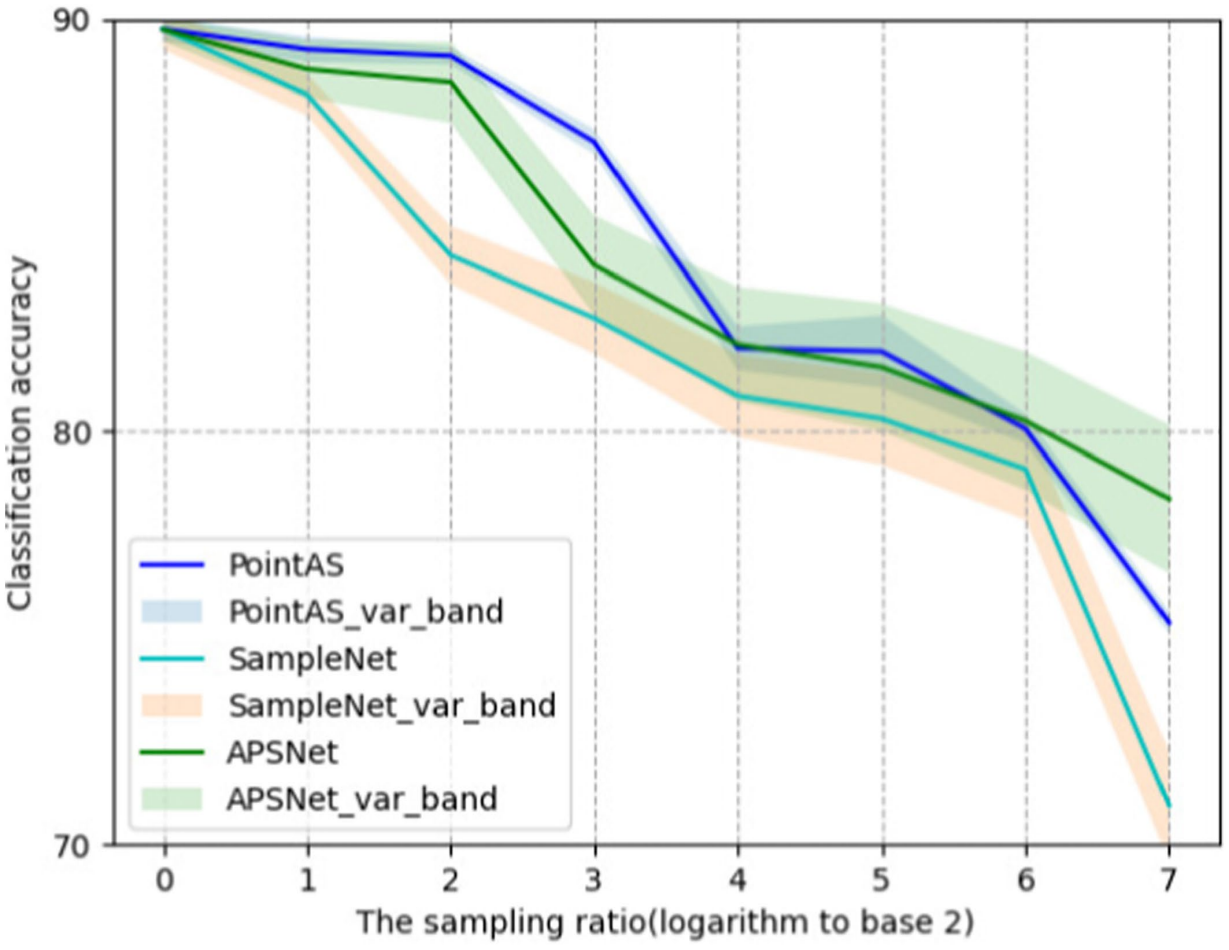
Demonstration of variance bands for the three methods PointAS, SampleNet and APSNet.

Figure 5 shows the ROC curves of different methods on ModelNet40 with a sampling rate of *m* equal to 512 (sample rate = 1), and the results show that the method in this paper occupies a larger area and is more effective. Figure 6 presents a performance comparison among six distinct sampling methods, from which qualitative insights emerge. As the sample size m increases (resulting in a lower sampling ratio), the accuracy of all sampling methods demonstrates improvement. Task-oriented samplers like SampleNet and APSNet consistently outshine their task-independent counterparts such as random sampling and FPS. However, the magnitude of gains gradually diminishes as m increases, with the accuracy curves showing a gradual plateauing effect as the sample rate decreases. Additionally, Figure 7 illustrates the statistical variances among PointAS, SampleNet, and APSNet. The solid lines represent the average performance across multiple experiments, while the shaded regions indicate the range of results from individual experiments. From the figure, it is evident that PointAS exhibits a more consistent and robust performance compared to SampleNet and APSNet. This suggests that PointAS is less sensitive to variations in experimental conditions and maintains a more stable performance across different trials.

When *m* equals 512 (sample rate = 1), all sampling methods approach an accuracy level closely resembling the optimal accuracy achieved with 1,024 points. Conversely, as ‘*m*’ decreases, the accuracy of RS, FPS, and S-Net for the classification task significantly deteriorates due to their limited adaptability to downstream tasks. In contrast, the enhanced SampleNet and APSNet demonstrate a more substantial degree of performance improvement for the classification task. Notably, PointAS consistently outperforms APSNet and SampleNet, particularly at a sampling rate of 3 (*m* = 128). Here, we observe a more substantial accuracy improvement, affirming the effectiveness of PointAS. Through Figure 7 we can see that PointAS coincides with the other methods in decreasing accuracy as the downsampling rate scales up, this is because as the downsampling rate becomes higher, the number of sampling points becomes smaller and the high-dimensional feature information that can be utilized plummets, which would be unfavorable for the classification task. However, PointAS is undoubtedly successful in terms of the magnitude of the accuracy reduction, as the magnitude of the reduction is small compared to other methods, indicating the effectiveness of the method in this paper.

**Figure 8:**
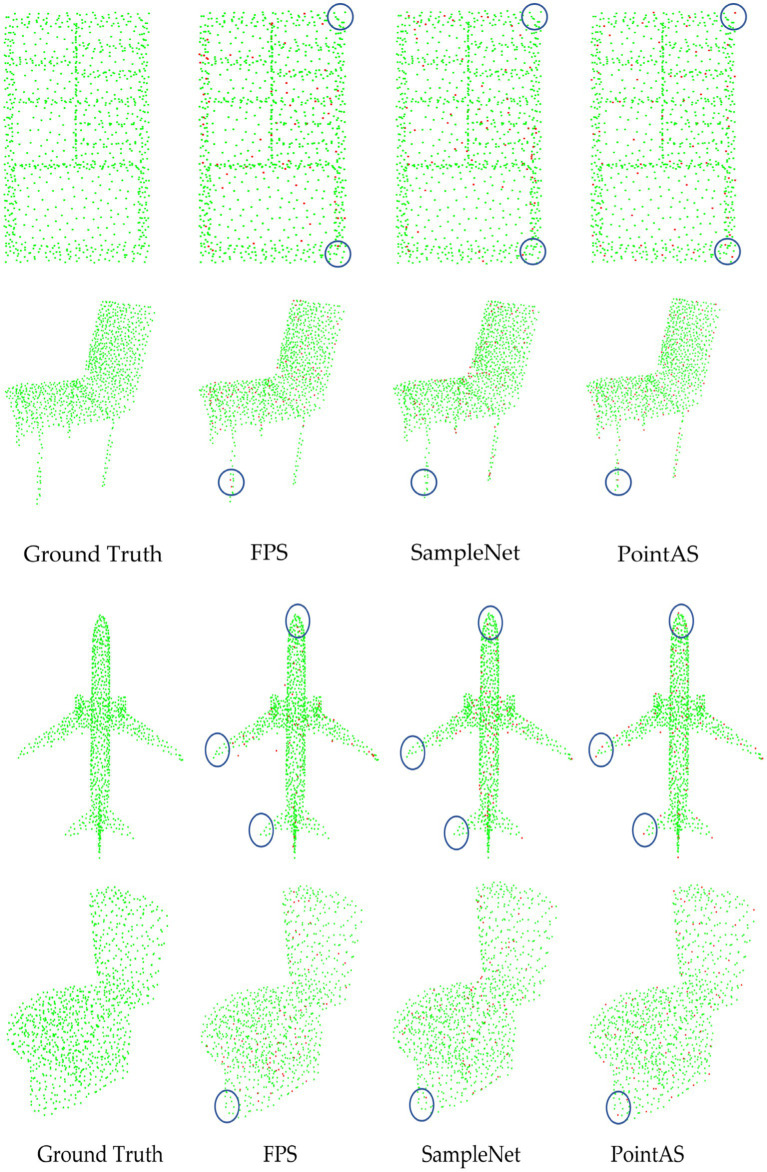
Visualization of downsampling data from different methods.

To visually illustrate the effectiveness of our model, Figure 8 provides downsampled views of cabinet, chair, toilet, and aircraft data using FPS, SampleNet, and PointAS. In the visual representation, green points denote the original data, while red points signify the downsampled data obtained through the respective methods. Upon comparison, it is evident that FPS and SampleNet struggle to accurately extract the four corners of the cabinet, as well as some other corner features. In contrast, PointAS excels in capturing crucial data features, particularly corners. Regarding the four feet information of the chair, both FPS and SampleNet nearly discard these features entirely, resulting in a decline in classification accuracy. As the sampling rate increases (and sample size *m* decreases), the absence of these crucial data features leads to a significant drop in classification accuracy. This effect is particularly pronounced in datasets like airplanes, where distinct features such as the nose, wings, and tail are prominent. The limitations of traditional methods become even more evident, as even the enhanced SampleNet struggles to extract essential sampling points like wings and tails. Conversely, PointAS consistently excels in extracting key point data, whether it pertains to cabinets, chairs, toilets, or aircraft, courtesy of its attention sampling module. Additionally, for diverse types of data, PointAS employs its adaptive module to precisely sample essential features. This adaptive approach stands as a pivotal factor contributing to a substantial improvement in sampling accuracy.

### 5.3 Noise immunity

To evaluate the resilience of our network in the presence of noisy inputs, we conducted two robustness tests. The noise in the experiment is added random Gaussian noise, i.e., random noise obeying Gaussian distribution is added to the original signal. This kind of noise can simulate many random perturbations that exist in real-world scenarios, such as sensor measurement errors, communication channel interference, etc. In these assessments, a predetermined level of noise was introduced to the original inputs. In the initial experiment pertaining to noise immunity, random noise within the range of [−1, 1] was added to all input point cloud data. To emphasize the significance of the experimental outcomes, we incorporated a tunable hyperparameter (denoted as σ ) into the noise input. By regulating the magnitude of σ , we could observe the algorithm’s robustness under varying levels of noise influence. Throughout all approaches, the input point count remained fixed at 1,024, while assumed values of 0.005, 0.01, and 0.03, respectively, for the comparative testing.

The quantitative results of the first robustness test are presented in Table 3, which unequivocally demonstrate the network’s proficiency in generating more condensed sampling results. Specifically, other sampling methodologies were markedly affected by the ambient noise, resulting in significant deviations in the positions of the sampled points. In contrast, PointAS model demonstrates remarkable classification accuracy even when subjected to varying levels of noise. This outcome affirms that our network effectively mitigates the impact of noise, ensuring that the sampled points retain the core characteristics of the objects.

**Table 3:** Classification accuracy with different noise disturbances.

*σ*	RS	FPS	S-Net	APSNet	SampleNet	PointAS
0.03	60.52	65.32	66.48	68.73	68.84	72.50
0.01	74.29	76.25	78.82	79.54	80.51	82.74
0.005	83.42	84.32	85.24	85.27	84.11	87.54
0	87.52	88.34	87.80	88.78	88.16	89.26

**Figure 9:**
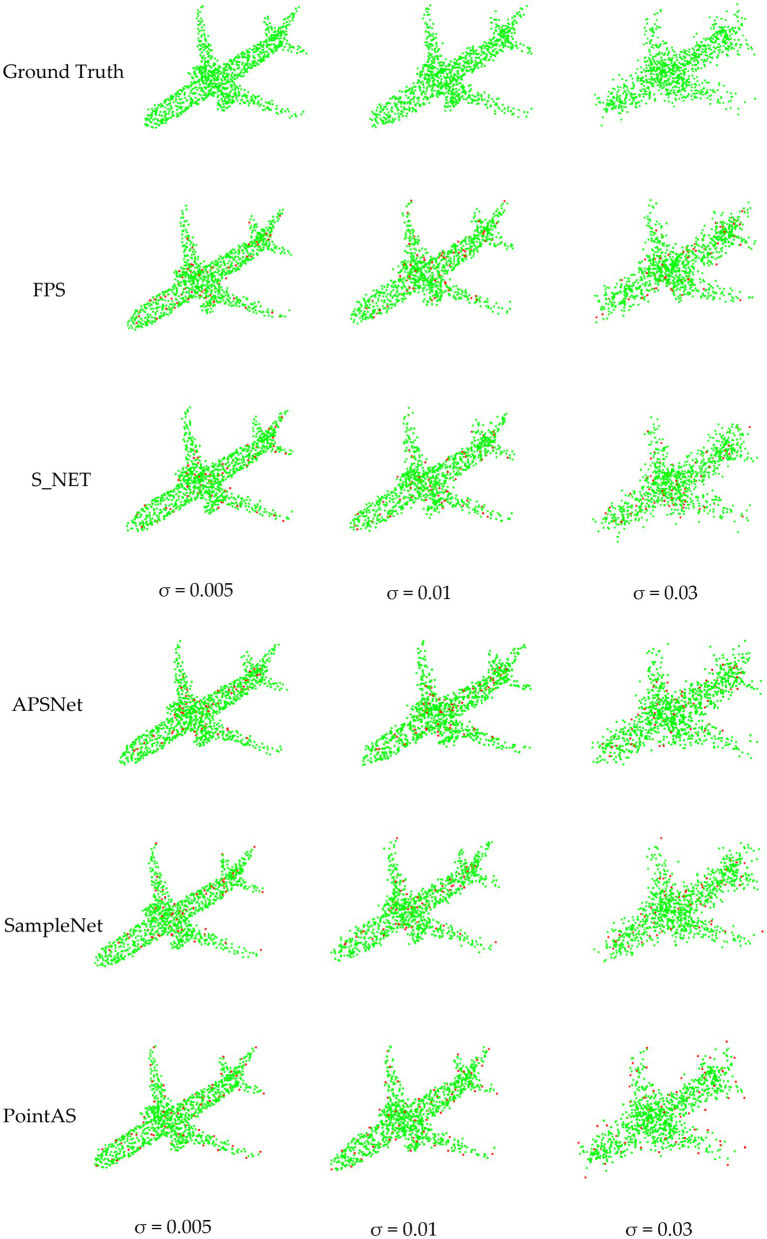
Comparison of point cloud downsampling results based on different noise disturbances.

The visualization of the first robustness test is depicted in Figure 9, showcasing the network’s proficiency in generating more streamlined sampling outcomes. Upon exposure to varying degrees of noise, APSNet exhibits a tendency to cause the disappearance of sharp features within the point cloud during the final sampling process. This is attributed to the neglect of local feature information, with the network primarily focusing on global information in its composition. As a result, the sampled point cloud exhibits defective corners, which is detrimental to subsequent classification tasks. SampleNet, on the other hand, effectively retains sharp features owing to its soft projection module. However, it encounters challenges in maintaining uniformity across the entire point cloud. This limitation arises from the consideration of only local factors in the overall sampling process, without incorporating global features for downstream tasks into the network computation. In contrast, the proposed method in this paper adeptly aggregates local feature points through the adaptive sampling module, enabling the retention of points with sharp features, such as those along the contour. Additionally, the integration of a global attention module ensures a certain degree of homogeneity across the entire point cloud, thereby yielding superior results.

Notably, noise has a negligible effect on the performance of PointAS, showcasing strong resistance to interference in point cloud downsampling. This characteristic is highly beneficial in object recognition and classification tasks. It allows for the discrimination of pertinent information from spurious data points, ultimately enhancing the performance of computer vision and machine learning algorithms in various domains, including robotics and security systems.

Another noteworthy advantage lies in the preservation of crucial geometric features. Competent downsampling methods ensure that significant details are retained, facilitating accurate measurements and precise 3D reconstructions. This is of paramount importance in fields such as architecture, archaeology, and civil engineering, where meticulous modeling and analysis are essential.

In our second series of anti-noise experiments, our primary goal is to assess the model’s responsiveness to noise. The process of identifying noise in point cloud downsampling employs advanced algorithms to analyze data point characteristics. These methods effectively identify and filter out noisy points by evaluating attributes like point density, outlier behavior, and statistical anomalies. This is especially crucial in fields such as lidar-based environmental sensing, where sensor noise, reflections, and moving objects can introduce inaccuracies. Additionally, downsampling methods that identify noise play a pivotal role in improving the precision of 3D object recognition and classification. Through the removal of spurious data points, these techniques guarantee that only meaningful information is preserved, thus enhancing the performance of computer vision and machine learning algorithms. This is particularly relevant in domains like autonomous navigation, where reliable data is paramount for safe and efficient operation.

We randomly select a specific percentage of points from the input, determined by *ω*, and subsequently introduce random noise within the range of [−1.0, 1.0]. In all approaches, the input point count remains constant at 1,024, with a downsample size of 64. To quantitatively illustrate the model’s sensitivity to noise, we compare the illustrations by computing the rate of noise in the final downsampling, which can be calculated in Eq. 14:


(14) $$\mathrm{Rate}=\frac{\mathrm{Nu}m_{\mathrm{noise}}}{1,024}$$

A lower Rate value indicates a higher sensitivity of the model to noise. The specific experimental results are presented in Table 4.

**Table 4:** Classification accuracy with different proportions of noise addition.

*ω*	RS	FPS	S-Net	APSNet	SampleNet	PointAS
0.1	0.25	0.11	0.21	0.09	0.19	0.05
0.2	0.32	0.16	0.31	0.16	0.29	0.09
0.3	0.42	0.21	0.37	0.24	0.36	0.14

From the quantitative analysis of Table 4, it is evident that PointAS exhibits a notable sensitivity to noisy inputs. Across different levels of noise intrusion, PointAS consistently ranks highest in sensitivity, with its highest rate value not exceeding 0.14. Even in cases of low perturbation, it can reach as low as 0.05, underscoring PointAS effective noise recognition capabilities. In contrast, the performance of other algorithms is comparatively weaker. They show varying degrees of sensitivity to different noisy inputs, which can be detrimental to downstream classification tasks and may adversely affect the outcomes of such tasks.

**Figure 10:**
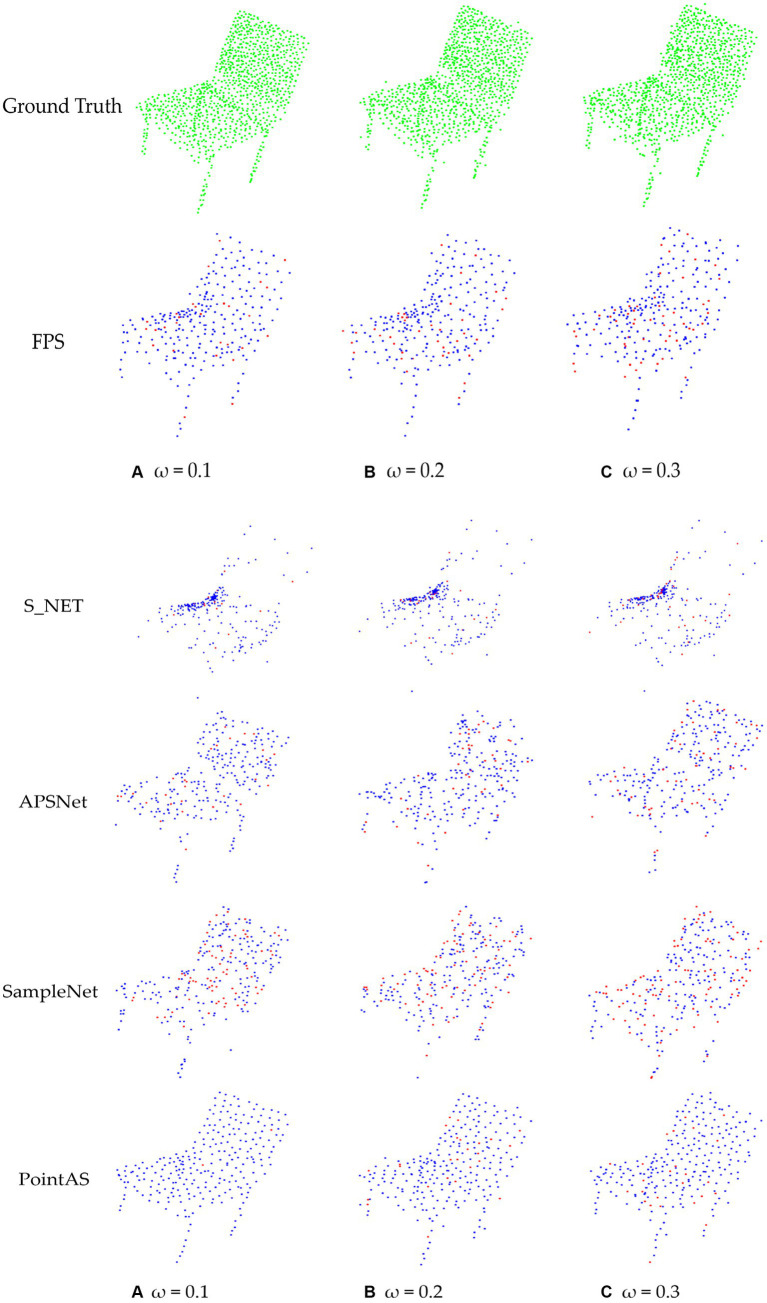
Comparison of point cloud downsampling results based on different proportions of noise addition: **(A)** Noise results with a scale of 0.1 added to the raw data; **(B)** Noise results with a scale of 0.2 added to the raw data. **(C)** Noise results with a scale of 0.3 added to the raw data.

The specific visualization results are depicted in Figure 10. Upon observation of Figure 9, it is apparent that the results obtained through PointAS downsampling exhibit a uniformly textured density, with noise exerting a negligible impact. Preserving geometric details is another key advantage of PointAS. It ensures that crucial information, such as object shapes and surface characteristics, is not lost during the downsampling process. This is indispensable for tasks like object recognition and precise measurements, where even minor details can be of great significance.

### 5.4 Ablation experiment

In this experiment, we primarily examine the impact of the adaptive sampling module and attention module on overall classification performance. To maintain simplicity, we set the downsampling rate *m* to 128 (sample rate = 1) and focus on assessing the improvement in accuracy achieved by each module individually.

**Figure 11:**
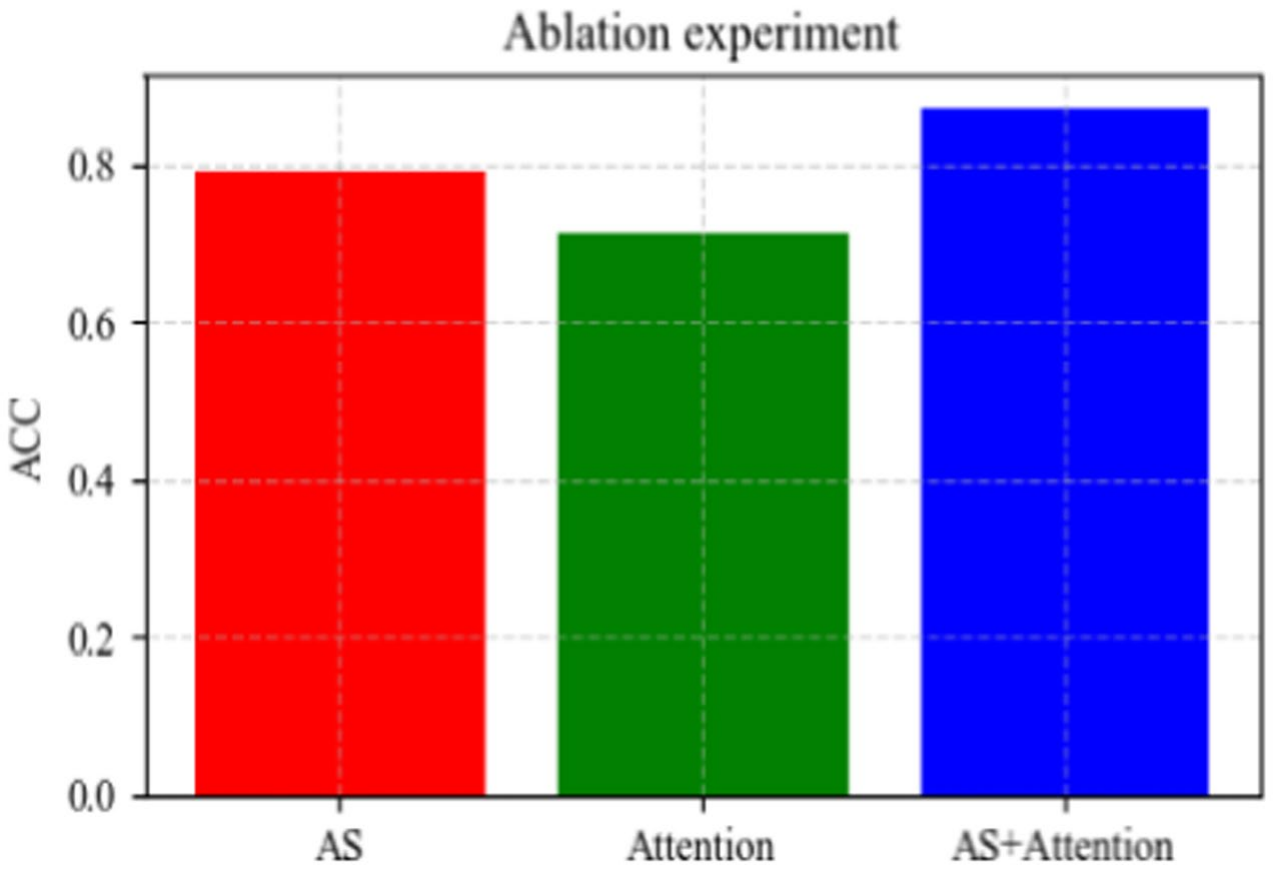
Comparison chart of ablation experiments.

The results, depicted in Figure 11, indicate that employing the adaptive sampling module alone yields better results compared to utilizing the attention module alone. As previously mentioned, the adaptive sampling module excels in aggregating neighborhood features to integrate local information effectively. On the other hand, the attention module focuses on integrating global information. However, relying solely on global information without local aggregation may result in minimal improvement in overall sampling accuracy. Conversely, combining both modules enables the aggregation of neighborhood information for integration with global information, leading to significant overall improvement. As illustrated in Figure 11, this combination results in a notable 6% increase in accuracy.

## 6 Conclusion

This paper introduces PointAS, comprising two primary modules: Attention Sampling and Adaptive Sampling. The input point cloud data is aggregated with global features through the attention module. Subsequently, the adaptive module extracts local features directly from these points. This combination of global and local sampling helps to improve the performance of the whole model. In the Adaptive Sampling module, an adaptive offset is generated for each neighborhood. Meanwhile, the Attention Sampling module utilizes a sequential autoregressive generation model to incorporate global information from the input data, allowing for the generation of downsampling points tailored for downstream tasks. In comparison to other downsampling algorithms used in point cloud classification tasks, PointAS may experience some reduction in accuracy, especially at high downsampling rates due to the adaptive sampling module. Nevertheless, PointAS exhibits distinct advantages in sample quality and inference speed, rendering it widely applicable across various practical scenarios. Furthermore, when applied to large-scale datasets, PointAS consistently demonstrates excellent performance. However, the supervised training of PointAS requires a large amount of data, and future developments may explore semi-supervised approaches. Additionally, further research could explore the application of PointAS to additional point cloud tasks such as reconstruction, detection, and segmentation, and enhance PointAS using advanced attention techniques.

## Data availability statement

The datasets presented in this study can be found in online repositories. The names of the repository/repositories and accession number(s) can be found at: http://modelnet.cs.princeton.edu/.

## Author contributions

BQ: Methodology, Software, Validation, Writing – original draft. SL: Supervision, Writing – review & editing. LW: Writing – review & editing.
